# TD-GC-MS Investigation of the VOCs Released from Blood Plasma of Dogs with Cancer

**DOI:** 10.3390/diagnostics3010068

**Published:** 2013-01-16

**Authors:** Roman Selyanchyn, Takuma Nozoe, Hidetaka Matsui, Tsuyoshi Kadosawa, Seung-Woo Lee

**Affiliations:** 1 Graduate School of Environmental Engineering, the University of Kitakyushu, 1-1 Hibikino, Wakamatsu-ku, Kitakyushu 808-0135, Japan; E-Mails: romanselyanchyn@gmail.com (R.S.); s1maa007@eng.kitakyu-u.ac.jp (T.N.); 2 Shinkou Seiki Co. Ltd., 1-18-3, Maidashi, Higashi-ku, Fukuoka 812-8581, Japan; E-Mail: matsui@shinkouseiki.co.jp; 3 Department of Veterinary Pathology, School of Veterinary Medicine, Rakuno Gakuen University, 582, Midorimachi, Bunkyodai, Ebetsu 069-8501, Japan; E-Mail: kado@rakuno.ac.jp

**Keywords:** dog blood plasma, TD-GC-MS, volatile organic compounds, cancer markers

## Abstract

An analytical TD-GC-MS method was developed and used for the assessment of volatile organic compounds (VOCs) released from the blood plasma of dogs with/without cancer. VOCs released from 40 samples of diseased blood and 10 control samples were compared in order to examine the difference between both sample groups that were showing qualitatively similar results independent from the disease’s presence. However, mild disturbances in the spectra of dogs with cancer in comparison with the control group were observed, and six peaks (tentatively identified by comparison with mass spectral library as hexanal, octanal, toluene, 2-butanone, 1-octen-3-ol and pyrrole) revealed statistically significant differences between both sample groups, thereby suggesting that these compounds are potential biomarkers that can be used for cancer diagnosis based on the blood plasma TD-GC-MS analysis. Statistical comparison with the application of principal component analysis (PCA) provided accurate discrimination between the cancer and control groups, thus demonstrating stronger biochemical perturbations in blood plasma when cancer is present.

## 1. Introduction

A large number of biochemical reactions proceed with great accuracy inside the body of live organisms. Nearly all these processes that are catalyzed by enzymes are subject to carefully controlled regulatory mechanisms inside the cells. In a normal, healthy state, therefore, the concentration of many different metabolites in blood is fairly constant [1]. However, it is believed that if biochemical processes become defective, usually due to a disease, the concentration of normal metabolites may change considerably, and in some cases new, abnormal metabolites may also be generated, usually defined as disease biomarkers [2]. The past decade has witnessed rapid progress in the understanding of the molecular basis of human illness, and it is expected that cures will be found for the major fatal diseases, particularly cancer [3]. More than four decades ago, Jellum *et al.* proposed that if one was able to identify and determine the concentration of all metabolites excreted by the human body, including both high and low molecular weight substances, one would likely find that many diseases would consequently result in characteristic changes of the biochemical composition of the cells and body fluids [2].

Cancer (tumor) markers are substances associated with cancer and appear in the human body when the disease is present. Appropriate measurement or identification of biomarkers is useful in patient diagnosis or clinical management. Tumor markers are most often found in the blood or urine, but they can also be found directly in tumors or other tissues. Markers can be produced by cancer cells themselves, or made by the organism in response to cancer [4]. The majority of currently known tumor markers in humans are proteins, namely the antigens associated with specific malignancies. However, with the exception of prostate-specific antigen (PSA), modern tumor markers are not useful for screening (early detection) purposes and are mainly used for monitoring the response to therapy and for detection of early relapse. According to a recent review, even PSA does not have sufficient sensitivity or specificity; its screening does not reduce mortality rates from prostate cancer [5]. Taking into account the efficacy of currently used cancer biomarkers, the studies of new cancer markers are of great importance.

Many research efforts are currently undertaking the search for new disease biomarkers (especially for cancer) among the small metabolites extracted from tissue [6], breath [7,8,9], breath condensate [10], urine [11,12], blood or other bodily fluids [13,14]. Fundamental researches also involve experiments to establish which volatile organic compound (VOC) can be specifically released or consumed by cancer cell cultures [15,16,17], or, alternatively, which biomarkers are released as a response to VOC exposure [18]. These studies are inspired by the successful results obtained in similar tests with other organisms such as algae [19] and bacteria [20], and their identification based on the exhaled VOC patterns. Non-protein markers including VOCs are usually called molecular markers, and are most commonly detected by hyphenated chromatographic techniques such as gas-chromatography mass spectrometry (GC-MS), liquid chromatography-mass spectrometry (LC-MS) [21], high performance liquid chromatography-MS (HPLC-MS) [22], HPLC with UV detection (HPLC-UV) [23], among others, while there have recently been many attempts to apply more simple devices such as electronic noses to this task [24,25]. In spite of chromatographic techniques offering a high level of specificity and sensitivity unrealized by spectrophotometric- and immunoassay-based methods, there are still many methodological problems with VOCs detection in most human samples, and most approaches are not the fast and simple analytical methods required by clinical practice [26,27].

Cancer specific VOCs extracted from human blood, serum or plasma have been found in several studies. Deng *et al*. reported the increase of heptanal and hexanal in the blood of lung cancer patients [19,20], and a recent approach by Xu and Wang proved that the same VOCs can be elevated in human serum [28]. However, to the best of our knowledge, large-scale studies in humans have not been conducted yet, as such studies necessitate quite strict regulatory and ethical control.

Dog models have been employed in numerous studies as an acceptable stage of research before moving to human research, once the methodologies are thoroughly tested. The similarities of some cancers between humans and canines, such as osteosarcoma, with regard to its histology, biological behavior and molecular genetic alterations, suggest that dogs provide a supplementary model for the development, and preclinical testing of, novel therapeutics [29]. In the study of Bentley *et al*., it was shown that bone marrow-derived hematopoietic cells appear to contribute to tumor angiogenesis in dogs, as it has been previously reported in humans, and that the biomarkers of angiogenesis not specific to tumor type have great potential for the objective assessment of treatment response [30]. A similar conclusion was made in the study of Uva *et al*., who compared genetic signatures of dog and human breast cancer samples: a close interspecies similarity in the network of cancer signaling circuitries in human breast cancer are largely maintained in the canine models [31]. Furthermore, many other studies confirm that biomarker studies in dogs are important not only for veterinary issues, but also for their potential value as “bridging biomarkers” for human diseases [32].

In this study, we analyzed the VOCs that can be evaporated from the blood plasma and pre-concentrated in an adsorption tube with Tenax GR adsorbent when active drying with pure helium stream is applied. The main task was the development of a GC-MS method for recognition between the blood plasma VOC profiles of dogs with cancer and healthy controls. An analysis of the obtained chromatograms was conducted with statistical methods, particularly non-parametric tests and principal component analysis (PCA) in order to distinguish differences between groups. To the best of our knowledge, this is the first study representing TD-GC-MS analysis of dog blood plasma VOCs as potential biomarkers of cancer independent of the disease. 

## 2. Experimental Section

Peripheral venous blood (2 mL) was obtained from 50 dogs during the routine visits to private veterinary hospitals. All blood samples were drawn into ethylenediaminetetraacetic acid (EDTA) tubes. Blood plasma was separated immediately by centrifugation for 15 min at 3,000 rpm. 0.5 mL of blood plasma was separated to an Eppendorf tube and refrigerated at 4 °C. The described procedure is standard for blood plasma collection and does not assure all VOC preservation in the sample. However, all samples were prepared in the same way to exclude significant influence of the procedure on the blood plasma VOC content. Samples were processed at two sites: Department of Veterinary Pathology, School of Veterinary Medicine, Rakuno Gakuen University, and Fukuoka animal hospital (CRICS Co. Ltd.), and finally transported to the GC-MS laboratory in the University of Kitakyushu. In the latter site, blood plasma samples were received on dry ice and transferred directly to the freezer with a temperature of −20 °C. Samples were collected from 40 dogs suffering from 23 different types of neoplasm, including 13 samples from dogs with solid malignant carcinoma, 9 samples—solid malignant sarcoma, 9 samples—hematological malignancies, 4 samples—melanoma, 1—mesothelioma, 3—benign tumors and 1 from the dog with inflammatory lesions (suspected cancer). 10 samples were received from healthy dogs and used as the control group. Details of all dogs’ characteristics, such as breed, age, sex and tumor type, can be found in Table 1. Due to the geographical separation between the collection and analysis sites, there was no possibility to analyze all samples immediately after collection, though all samples were kept frozen and thawed directly before analysis. 

**Table 1:** Samples description data: number, breed, age, sexual status, type of tumor.

Healthy group		n	Cancer group	n
**Breed**	Mixed breed	2	Mixed breed	11
	Chihuahua	2	Golden Retriever	6
	Poodle	1	Labrador Retriever	5
	Pomeranian dog	1	Beagle	3
	Toy poodle	1	Miniature dachshund	3
	Miniature dachshund	1	French Bulldog	2
	Wire Fox Terrier	1	Shetland Sheepdog	2
	Welsh Corgi Pembroke	1	Basset Hound	1
			Bearded Collie	1
			Curly Coated Retriever	1
			English Cocker Spaniel	1
			Pomeranian	1
			Pug	1
			Shih Tzu	1
			Toy poodle	1
**Sex**	Female (intact)	8	Female (intact)	9
	Female (spayed)	1	Female (spayed)	12
	Male	1	Male (intact)	13
			Male (castrated)	6
**Age**	mean ± SD	5.7 ± 3.1 years	mean ± SD	9.2 ± 2.6 years
	median	5.3 years	median	9.5 years
	range	2 to 13 years	range	2 to 18 years
			**Tumor type**	
			Benign	3
			*(among them)*	
			- mammary gland tumor	2
			- inflammatory polyp	1
			Malignant	36
			*(among them)*	
			- solid tumor, carcinoma (10 types)	13
			- solid tumor, sarcoma (6 types)	9
			- hematological malignancies (2 types)	9
			- melanoma	4
			- mesothelioma	1
			Other	1
			- inflammatory lesions (susp. cancer)	1

Tenax GR, mesh size 60–80, pre-packed quartz glassy tubes were purchased from Japan Analytical Industry (JAI Co. Ltd., Japan), glassy wool was purchased from GL Sciences (Japan), and deionized water was obtained by reverse osmosis in Milli-Q system. In the commercial quartz tube, 20 mg of Tenax GR was wrapped by a ferromagnetic foil and thermal desorption of VOCs from adsorbent was performed by the use of Curie point effect in a JCI-22 Curie point pyrolyzer device (JAI Co. Ltd., Japan). 

**Figure 1:**
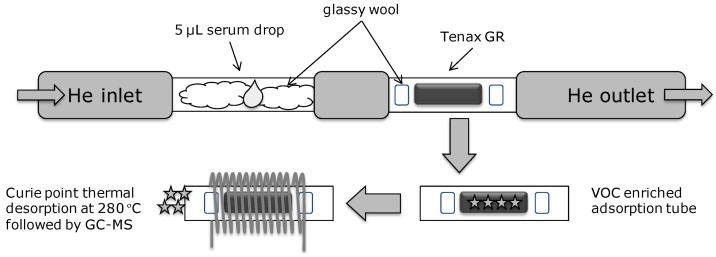
Schematic illustration of sample preparation before injection into the gas-chromatograph (GC).

A simple sampling method was applied, and includes the following steps. At first, glassy tubes are thoroughly washed in deionized water with sonication and dried in nitrogen. Then, small pieces of glassy wool are wrapped into a cleaned pyrofoil of a certain temperature, inserted into the prepared quartz tubes, and baked twice in the Curie point injector (heating to 150 °C followed by pyrofoil temperature, 280 °C heating for 15 s) for cleaning purposes. Double baking was also performed for the purchased Tenax GR tubes before sampling to remove the possible contaminants. When two tubes—one with glassy wool and the other with adsorbent—were ready, blood plasma samples were thawed from −20 °C to the room temperature and shaken for 1 min using a mini-shaker (MS1 Minishaker, IKA Ltd.) at 1,400 rpm, in order to homogenize the sample. After shaking, 5 μL of blood plasma was immediately taken by using a 25 μL Hamilton syringe and transferred inside the glassy wool (scheme of the sample collection is shown in Figure 1). Then both tubes are connected by the use of a silicon tube and the blood plasma sample is dried in 50 mL/min of extra-pure helium flow for 10 min through the Tenax GR column. Both tubes were maintained at room temperature. As has been checked in the preliminary test by observing related mass changes, such procedure leads to complete sample drying. All samples were tested in duplicate in order to assess repeatability of the GC-MS analysis. CPI injection of collected VOCs into the Tenax GR column was performed at 280 °C for 15 s without purge or pre-heating in a JCI-22 pyrolyzer. 

Chromatographic analysis with mass spectrometric detection was carried out with Jms-Q1000GC (JEOL, Japan) GC-MS system consisted of an Agilent 7890A GC coupled to a quadrupole MS. The injector and ion source temperatures were kept at 230 °C. GC oven temperature was kept at 40 °C for 3 min, increased at 3 °C/min to 110 °C where it was held for 2 min and further increased at 10 °C/min to 230 °C where it remained for 20 min. Extra-pure He gas (99.99995%, Air Liquid Kogyo Gas Ltd., Japan) was used as mobile phase at a flow-rate of 1 mL/min. The analytical capillary column used for separation was a DB-WAX (polyethylene glycol based high-polarity stationary phase, 30 m length, 0.25 mm inner diameter, 0.5 μm film thickness, Agilent J&W, part number 19091J-413). Start cut-off time for MS recording was 3 min. The MS was operated in electron ionization (EI) mode at 70 eV. Data acquisition was performed in the full scan mode from m/z = 25–310 with a scan time of 0.3 s.

For every sample, freshly washed quartz tubes were used to avoid sample carryover and cross contamination. Chromatogram acquisition and tentative compound identification was done by the National Institute of Standards and Technology (NIST) mass spectral library search, performed using the JEOL (Version 1.0.3208.25600) software.

Statistical significance was assessed with the use of non-parametric Mann-Whitney U-test for each compound (implemented as function of Origin 8.0 data analysis package) to compare samples from two different groups for independent observations (in our case different VOC concentrations were determined as AUC in selected ion chromatogram). PCA was conducted with Microsoft Office Excel add-in (XLSTAT Version 2011.4.02) and used to describe the “total” difference between two groups. Selected ion AUCs for each VOC was used as PCA input variables. Normality tests for each compound distribution within two sample groups were also run using XLSTAT package.

## 3. Results and Discussion

### 3.1. Typical Chromatogram

In order to determine which VOCs evaporated from the sample during drying in the stream of He gas, blank (or reference) chromatograms were collected and compared with those of the samples. In our case, blank chromatograms are obtained by TD-GC-MS analysis of the Tenax GR adsorption tube, after blowing He gas at the same flow rate of 50 mL/min for 10 min without sample. 

**Figure 2:**
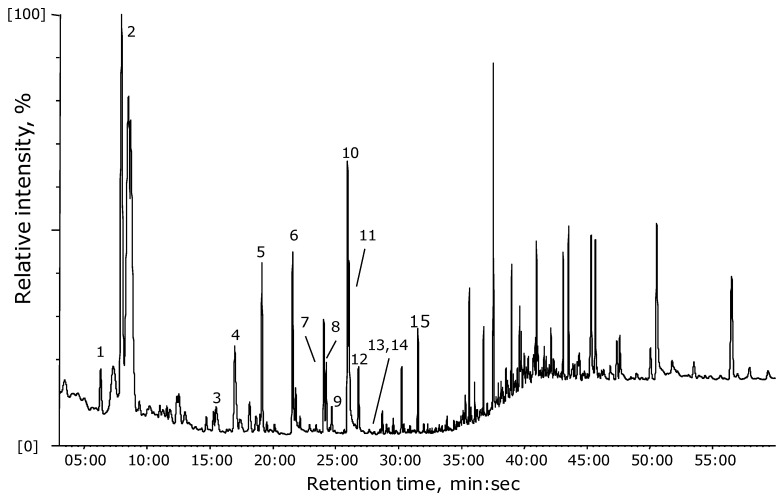
Typical (selected ions) chromatogram of volatile compounds released from 5 μL of blood plasma during drying in pure He gas. VOCs collected in a small sorption tube filled with Tenax GR and subsequently thermally desorbed into the GC-MS system by the means of Curie point flash pyrolysis. Ions m/z = 43, 44, 56, 57, 60, 67, 72, 80, 84, 91, 104, 105, 106, and 108 used for representation.

In spite of extra-pure He gas with a purity of 99.99995% being used for sample drying, various peaks appear on the blank chromatogram, evidencing the capture of He contaminants in the adsorbent. Before the analysis, all tubes with adsorbent were treated according to the procedure recommended by the supplier (*i.e.*, twice time baking in the CPI device). This procedure was sufficient to clean up the tubes; it was further confirmed by GC-MS test. However, considering the fact that in our case the blank chromatogram was obtained when helium was blown through the adsorption tube for 10 min at 50 mL/min flow, in total, contaminants from 500 mL of He could be captured. The presence of impurities is not seen as chromatographic peaks during the routine run (He flow-rate was 1 mL/min), though it looks to be pre-concentrated in the adsorption tube. Thus we consider even pure He as the source of the contamination, with total impurity less than 0.5 ppm according to the He provider. Other parts of the sample collection (silicon tubes, fittings) can also contribute to the background, so for the blood plasma samples comparison, VOCs found in background have been skipped from analysis. Blank chromatograms have been accurately assessed in order to skip the compounds that are present in the drying gas, *i.e*., peaks present in the blank with SNR > 3 were not counted. A typical chromatogram in the representation of characteristic ions of the VOCs released from 5 μL of blood plasma is given in Figure 2. As given here by number labels, only 15 VOCs originating from the small amount of dried blood plasma samples were clearly distinguished from the background (SNR > 10). Taking into account reference studies and the established fact that hundreds of different chemical compounds are present in blood or blood plasma, our current result reveals much less, but this amount is reasonable if one considers the small amount of sample used for VOC collection. Therefore, only VOCs with relatively high concentrations are detected, while the concentration of many other trace compounds lies below the detection limit of the experimental method. Nevertheless, enough data was collected to confirm the differences between groups. 

### 3.2. Repeatability of Chromatograms

The chromatograms of the several samples (five replicates) were checked for repeatability, and they were quite repeatable with a relative standard deviation (RSD) less than 20% for each reported compound. However, when the sample was assessed after a three-month interval, significant changes in the chromatogram were observed, and the number of compounds also decreased. This means that certain samples’ loss of the VOC content occurs while thawing/refreezing the sample. For instance, for aldehydes such as nonanal and octanal, the change of intensity with time was estimated about 30% after a few months and a second thawing cycle. According to the WHO regulations of blood plasma storage [33], fresh-frozen plasma can be stored at the temperature −20 °C for at least five years without significant loss of integrity. However, repeated thawing and freezing may cause denaturation of plasma constituents. While there are no regulations regarding VOC content of blood plasma, we followed the WHO standards for blood plasma processing. Thus all samples in our case were analyzed within one month after receipt and only after first thawing. In order for correct assessment of VOCs released from blood plasma with the described method, the samples should be analyzed as soon as possible after collection, and kept in appropriate frozen conditions before analysis. Moreover, taking into account that the solubility of VOCs depends on the sample temperature [34], and their concentrations extracted from fresh samples can differ from those frozen and thawed before analysis, we did not analyze fresh samples, but all samples were analyzed and compared after first thawing, *i.e.*, after the absolutely same processing history.

### 3.3. Compounds Released from Blood Plasma

Based on the comparison with the blank chromatogram (helium through the Tenax GR), the intensity of several compounds was found much higher than in the blank, or was not present in the blank at all. These compounds were considered to be VOCs evaporated from the blood plasma samples. As already mentioned, to assure that all compounds are well detected by the described method, only peaks with high signal-to-noise ratios (SNR > 10) were considered. A list of the detected VOCs with names tentatively identified by the NIST library, main ions found in mass spectra, retention times, and CAS registry numbers are given in Table 2, and also indicated by numbers in the chromatogram in Figure 2. These VOCs were subject to comparison between the healthy and cancer patients groups. Several normality tests (Shapiro-Wilk, Anderson-Darling, Lilliefors and Jarque-Bera) were run with all samples confirming that data acquired for all compounds do not follow normal distributions, thereby justifying the use of the non-parametric Mann-Whitney test for significant differences. In spite of the certain possibility of capturing volatiles from the blood plasma samples, the described GC-MS method does not account for all VOCs evaporated from the samples during drying, because of the limited possibilities of the sorbent (adsorption properties of Tenax GR) and GC capillary column (chemical properties of DB-WAX). However, this combination was best available in the laboratory at the time of experiment. Thus, in this study, we observed only 15 VOCs clearly distinguishable from the background, for which comparison between both sample groups have been performed. All these compounds’ GC data (peak areas) was used also for PCA, considering that they are common volatile metabolites for the blood plasma samples independently of disease presence even in normal conditions. This kind of comparison will give a possibility to assess “global,” general metabolic disturbance due to disease. 

**Table 2:** Volatile organic compounds, metabolites detected by the thermal desorption gas chromatography-mass spectrometry (TD-GC-MS) analysis of dog’s plasma samples for both investigated groups (identified by comparison of mass spectrum with NIST library).

#	Tentative compound name	Main ion, m/z	Retention time, min:sec	CAS number	Probable metabolic origin	Significance test *p-value*
1	Toluene	91	06:20	108-88-3	Exogenous exposure [35]	***0.008***
2	Hexanal	56	08:50	66-25-1	Natural metabolites, mediators of oxidative stress [36]	***0.008***
3	Styrene	104	15:10	100-42-5	Exogenous exposure but also naturally present at few ppb levels [37]	*0.692*
4	Octanal	43	16:50	124-13-0	*Same as #2.Hexanal*	***0.002***
5	5-Hepten-2-one, 6-methyl-	43	19:02	110-93-0	Natural VOC, exogenous [38]	*0.477*
6	Nonanal	57	21:30	124-19-6	*Same as #2.Hexanal*	*0.934*
7	Acetic acid	60	24:00	64-19-7	Normal metabolite [36]	***0.205***
8	1-Octen-3-ol	57	24:11	3391-86-4	Natural production VOC for simple mushrooms [39]	***0.001***
9	2-Butanone	43	24:36	78-93-3	Natural product found in plants [36], potential disease marker	***0.005***
10	1-Hexanol, 2-ethyl-	57	25:51	104-76-7	Widely used fragrance ingredient, occurring in nature [40]	*0.852*
11	Decanal	57	25:57	112-31-2	*Same as #2.Hexanal*	*0.131*
12	Benzaldehyde	106	26:41	100-52-7	Flavoring agent, occasionally found in urine [36]	*0.054*
13	Pyrrole	67	26:27	109-97-7	Naturally occurring compounds, could be relevant to diseases [41]	***0.029***
14	1H-Pyrrole, 3-methyl-	80	28:16	616-43-3		*0.054*
15	Acetophenone	105	31:27	98-86-2	Food additive	*0.441*

### 3.4. Influence of Demographic Variables

Several parameters characterizing the blood plasma samples including type of disease, dog breed type, age and sex were also available. Separate statistical assessment was undertaken in order to check the influence of these variables on the obtained results, though there were no statistically significant correlations between these parameters and concentrations (chromatographic peak areas) of compounds extracted from GC-MS data (data not shown). Disease influence was not assessed due to a wide variety of cancer types (23 disease types for 40 samples). Detailed information about the stage of the disease cancer site and histology was not available for the samples, and thus simple group division—cancer *vs.* control—was used for results presentation. Thus the main comparison was done for the criteria cancer *vs.* non-cancer without accounting for other patient parameters. Considering the reference studies, such comparison is valid due to the possibility of the existence of the biomarkers signaling the tumor angiogenesis or cancer-related oxidative stress in the organism. Such biomarkers are not specific to the particular disease, but are more likely to provide evidence of the intensification of the vascular growth and development in the area of the tumor. 

### 3.5. Statistical Comparison of the Two Groups

**Figure 3:**
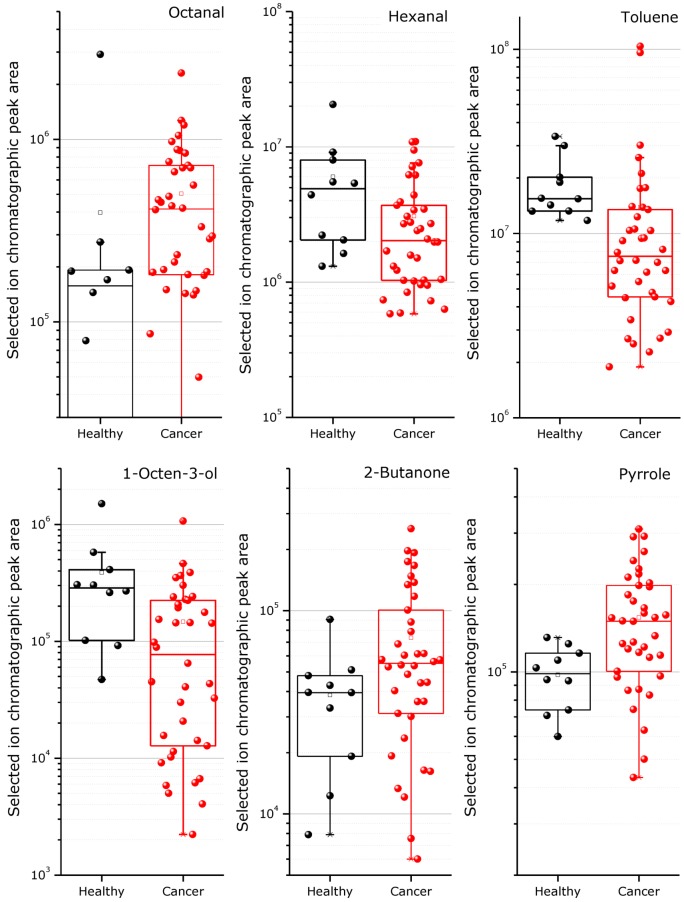
Distribution charts representing the variance in healthy and cancerous sample groups for six common volatile metabolites released from blood plasma during drying by He gas, subsequent adsorption in a Tenax GR filled tube and TD-GC-MS analysis (y-scale showing peak area in chromatogram, points represent individual measured samples, box borders, respectively, 25 and 75% samples fitting).

As stated previously, the non-parametric Mann-Whitney test is appropriate for assessing whether two independent samples of observations have different average values. It is one of the best non-parametric significance tests and is used similarly to the Student *t*-test without necessary requirement for samples to follow normal distribution. It was used for different variables’ (VOCs released from the blood plasma) comparison in cancer and control subject groups. Graphical distribution of concentrations for compounds with statistically significant differences (*p*-value < 0.05) from Table 2 is shown in Figure 3. From these charts, we can see that it is difficult to select volatile substances, which can uniquely indicate the cancer presence, from the investigated list. However, these differences for the respective compounds in the distribution charts are quite informative. Some of these compounds were already reported as potentially related to cancer in human studies. As an abnormal condition in a body cancer, it leads to cellular oxidative stress, and respectively, to the emission of cancer-specific VOCs into the blood [25,36,42,43]. 

The presence of several species in the blood (particularly toluene and 2-butanone) can result from environmental exposure; however, we did not possess the information about the sample dogs’ histories of exposure to any organic vapors. Even toluene is commonly reported as an exogenous compound for human excretions [35]; in the study of Poli *et al.* [44], its elevation was seen in patients with lung cancer in comparison with non-smoking healthy controls. On the other hand, an increase of 2-butanone in the breath of lung cancer patients was observed by Bajtarevic *et al*. [9]. In our case, however, we obtained the opposite result for toluene (decreases in cancer group), but the same result for the compound tentatively identified as 2-butanone (increased in cancer group similarly to [9]), as can be seen from the distribution chart in Figure 3 and from the result of the Mann-Whitney test. However, more detailed studies for selected disease conditions are needed to confirm the validity of all suggested biomarker compounds. 

Octanal and hexanal—two tentatively identified aldehyde compounds have also been often reported as VOC cancer biomarkers in human samples, as well as many other aldehydes [27]. In this study, both aldehyde compounds also show significant differences between groups: hexanal is oppositely decreased, while octanal is increased, similar to several human sample studies [13,27]. On the other hand, in the cell culture-based experiments, hexanal was found to be consumed by cancer cells in comparison with healthy ones [45]. Although both aldehydes were statistically different between the groups, the measurement of these compounds alone is unlikely to provide valuable clinical information.

The two remaining tentatively identified compounds (1-octen-3-ol and pyrrole) revealing statistical difference have rarely been reported as disease biomarkers. However, they can be relevant to the biological processes [39,41].

**Figure 4:**
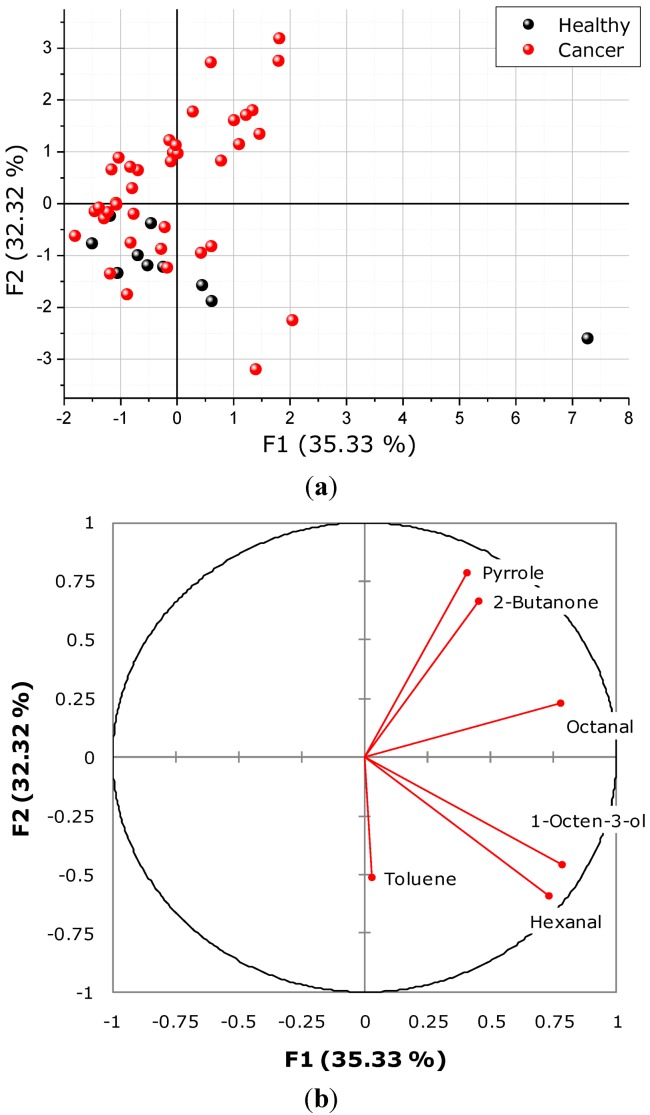
(**a**) PCA of the PC1 and PC2 values (F1 and F2) resulting from statistical analysis of the abundance of volatile organic compounds determined by TD-GC-MS analysis of dog plasma samples, using volatile compounds observed in both healthy and cancer samples. (**b**) loading chart indicating the contribution of the variables in total variability of first two principal components (F1 and F2).

Principal component analysis is a useful statistic approach to reduce total variability in the data to more informative principal components and can be used to investigate recognition between the samples with or without cancer. The data of six significantly different compounds observed for 50 samples in two groups were used as independent variables for PCA. The result of PCA is shown in Figure 4(a) for the first two principal components accounting for 67.7% of total variability of metabolites. As it can be seen from Figure 4, complete recognition between the control and cancerous groups was not achieved by PCA treatment of the obtained TD-GC-MS data. However, the PCA points related to the healthy samples (except for one sample) can be certainly grouped on the background of much wider distribution of the cancerous samples. This result is most likely significant evidence of biochemical changes in the blood plasma composition due to disease progression. Only one sample is significantly different from others in the healthy group; it possessed the VOC features that are also observed in cancerous samples, though it is probably related to some other condition and not to cancer. The obtained result confirms general molecular cancer markers that can be found in the literature, *i.e.*, there is strong evidence of cancer-related VOCs present in dogs with cancer plasma samples. However, no compounds have been found that can be used as pure, diagnostic decision-making cancer biomarkers. It is very important to continue this activity with variation in sample collection, preparation, data analysis, *etc.*, in order to confirm which compounds can serve as potential cancer biomarkers and proceed to the clinical trials with larger populations.

## 4. Conclusions

The current TD-GC-MS study aimed to investigate the biochemical perturbation of the blood plasma induced by cancer in dog samples in comparison with normal control subjects. Our study confirmed that specific changes in the metabolic composition of cancerous blood plasma take place in the case of cancer in dogs. In spite of certain limitations of the study—a variety of cancer samples in the population (different breeds and cancers), a small control group, and mere qualitative comparison without peak validation by standards—several VOCs demonstrated statistically significant difference in the two groups. Due to the variety of samples in respect to cancer type, it is at present difficult to claim the compounds specific to a certain disease. Additional investigations are needed in more narrow groups, preferably ones with the same disease and dog breed; this would clarify if the disease-specific VOC biomarkers could be found in blood plasma. However, tentative VOCs reported in our study can be considered as non-specific cancer biomarkers of cancer-related conditions, for example, enhanced angiogenesis or oxidative stress usually taking place in an organism with a tumor. Heterogeneity of the study population supports this conclusion.

Variance between both sample groups showed statistically significant difference for six peaks (tentatively identified by NIST library VOCs: hexanal, octanal, toluene, 2-butanone, 1-octen-3-ol and pyrrole) released from blood plasma during the drying process, with a level of significance *p* < 0.05. However, none of the detected VOCs can be used as a single diagnostic predictor—even an account of all the significant factors does not lead to the clear group separation when using PCA. 

Certain features of cancer and control sample distribution, namely, a much wider spread of data points related to the cancer samples in comparison with the relatively grouped healthy samples, concludes the evidence of the altered VOC production by an organism affected by cancer. 

Obtained results demonstrate that the described TD-GC-MS technique can be developed as an alternative tool for the characterization of the metabolic perturbation in blood plasma samples to provide recognition and prognosis of cancer diseases; however, proper identification by standards and quantitative validation of all results should be first conducted. Additionally, the usage of dog samples could provide an alternative analytical methodology before investigating human samples that are often stricken by regulatory and ethical issues. To the best of our knowledge, our current work is the first investigation of VOC cancer biomarkers in dogs, providing approximate identification based on the cancer/cancer-free criteria. However, considering the aforementioned limitations, this preliminary result should be verified by a larger study.

## References

[1] Rosenthal M.D., Glew R.H. (2009). Medical Biochemistry: Human Metabolism in Health and Disease.

[2] Jellum E., Stokke O., Eldjarn L. (1973). Application of gas chromatography, mass spectrometry, and computer methods in clinical biochemistry. Anal. Chem..

[3] Ma S., Turino G.M., Lin Y.Y. (2011). Quantitation of desmosine and isodesmosine in urine, plasma, and sputum by LC-MS/MS as biomarkers for elastin degradation. J. Chromatogr. B.

[4] Duffy M.J. (2001). Clinical uses of tumor markers: A critical review. Crit. Rev. Clin. Lab. Sci..

[5] Ilic D., O’Connor D., Green S., Wilt T.J. (2011). Screening for prostate cancer: An updated Cochrane systematic review. BJU Int..

[6] Wu H., Xue R., Lu C., Deng C., Liu T., Zeng H., Wang Q., Shen X. (2009). Metabolomic study for diagnostic model of oesophageal cancer using gas chromatography/mass spectrometry. J. Chromatogr. B.

[7] Amann A., Poupart G., Telser S., Ledochowski M., Schmid A., Mechtcheriakov S. (2004). Applications of breath gas analysis in medicine. Int. J. Mass Spectrom..

[8] Modak A.S. (2010). Breath biomarkers for personalized medicine. Personalized Med..

[9] Bajtarevic A., Ager C., Pienz M., Klieber M., Schwarz K., Ligor M., Ligor T., Filipiak W., Denz H., Fiegl M. (2009). Noninvasive detection of lung cancer by analysis of exhaled breath. BMC Cancer.

[10] Horváth I., Hunt J., Barnes P.J., Alving K., Antczak A., Baraldi E., Becher G., van Beurden W.J.C., Corradi M., Dekhuijzen R. (2005). Exhaled breath condensate: Methodological recommendations and unresolved questions. Eur. Resp. J..

[11] Banday K.M., Pasikanti K.K., Chan E.C.Y., Singla R., Rao K.V.S., Chauhan V.S., Nanda R.K. (2011). Use of urine volatile organic compounds to discriminate tuberculosis patients from healthy subjects. Anal. Chem..

[12] Dubbelman A.C., Tibben M., Rosing H., Gebretensae A., Nan L., Gorman S.H.,  Robertson P., Schellens J.H.M., Beijnen J.H. (2012). Development and validation of LC-MS/MS assays for the quantification of bendamustine and its metabolites in human plasma and urine. J. Chromatogr. B.

[13] Deng C., Li N., Zhang X. (2004). Development of headspace solid-phase microextraction with on-fiber derivatization for determination of hexanal and heptanal in human blood. J. Chromatogr. B.

[14] Deng C., Zhang X., Li N. (2004). Investigation of volatile biomarkers in lung cancer blood using solid-phase microextraction and capillary gas chromatography-mass spectrometry. J. Chromatogr. B.

[15] Sponring A., Filipiak W., Mikoviny T., Ager C., Schubert J., Miekisch W., Amann A., Troppmair J. (2009). Release of volatile organic compounds from the lung cancer cell line NCI-H2087 *in vitro*. Anticancer Res..

[16] Filipiak W., Sponring A., Mikoviny T., Ager C., Schubert J., Miekisch W., Amann A., Troppmair J. (2008). Release of volatile organic compounds (VOCs) from the lung cancer cell line CALU-1 *in vitro*. Cancer Cell Int..

[17] Sponring A., Filipiak W., Ager C., Schubert J., Miekisch W., Amann A., Troppmair J. (2010). Analysis of volatile organic compounds (VOCs) in the headspace of NCI-H1666 lung cancer cells. Canc. Biomarkers.

[18] Fischäder G., Röder-Stolinski C., Wichmann G., Nieber K., Lehmann I. (2008). Release of MCP-1 and IL-8 from lung epithelial cells exposed to volatile organic compounds. Toxicol. In Vitro.

[19] Laturnus F. (1995). Release of volatile halogenated organic compounds by unialgal cultures of polar macroalgae. Chemosphere.

[20] Allardyce R.A., Langford V.S., Hill A.L., Murdoch D.R. (2006). Detection of volatile metabolites produced by bacterial growth in blood culture media by selected ion flow tube mass spectrometry (SIFT-MS). J. Microbiol. Meth..

[21] Theodoridis G., Gika H.G., Wilson I.D. (2008). LC-MS-based methodology for global metabolite profiling in metabonomics/metabolomics. TrAC Trends Anal. Chem..

[22] Wilson I.D., Plumb R., Granger J., Major H., Williams R., Lenz E.M. (2005). HPLC-MS-based methods for the study of metabonomics. J. Chromatogr. B.

[23] Bullinger D., Fröhlich H., Klaus F., Neubauer H., Frickenschmidt A., Henneges C., Zell A., Laufer S., Gleiter C.H., Liebich H., Kammerer B. (2008). Bioinformatical evaluation of modified nucleosides as biomedical markers in diagnosis of breast cancer. Anal. Chim. Acta.

[24] Di Natale C., Macagnano A., Martinelli E., Paolesse R., D'Arcangelo G., Roscioni C., Finazzi-Agrò A., D’Amico A. (2003). Lung cancer identification by the analysis of breath by means of an array of non-selective gas sensors. Biosens. Bioelectron..

[25] Peng G., Hakim M., Broza Y.Y., Billan S., Abdah-Bortnyak R., Kuten A., Tisch U., Haick H. (2010). Detection of lung, breast, colorectal, and prostate cancers from exhaled breath using a single array of nanosensors. Brit. J. Cancer.

[26] Hammett-Stabler C., Garg U. (2010). The evolution of mass spectrometry in the clinical laboratory. Meth. Mol. Biol..

[27] Hakim M., Broza Y.Y., Barash O., Peled N., Phillips M., Amann A., Haick H. (2012). Volatile organic compounds of lung cancer and possible biochemical pathways. Chem. Rev..

[28] Xu H., Wang S. (2012). A novel sorptive extraction method based on polydimethylsiloxane frit for determination of lung cancer biomarkers in human serum. Anal. Chim. Acta.

[29] Rankin K.S., Starkey M., Lunec J., Gerrand C.H., Murphy S., Biswas S. (2012). Of dogs and men: Comparative biology as a tool for the discovery of novel biomarkers and drug development targets in osteosarcoma. Pediatr. Blood Canc..

[30] Bentley R.T., Mund J.A., Pollok K.E., Childress M.O., Case J. (2012). Peripheral blood biomarkers of solid tumor angiogenesis in dogs: A polychromatic flow cytometry pilot study. Vet. J..

[31] Uva P., Aurisicchio L., Watters J., Loboda A., Kulkarni A., Castle J., Palombo F., Viti V., Mesiti G., Zappulli V. (2009). Comparative expression pathway analysis of human and canine mammary tumors. BMC Genomics.

[32] Selvarajah G.T., Kirpensteijn J. (2010). Prognostic and predictive biomarkers of canine osteosarcoma. Vet. J..

[33] Requirements for the Collection, Processing and Quality Control of Blood, Blood Components and Plasma Derivatives; WHO Technical Report Series, No 840. http://www.who.int/bloodproducts/publications/WHO_TRS_840_A2.pdf.

[34] Lin J.-H., Chou M.-S. (2006). Henry’s law constant variations of volatile organic compounds in wastewater and activated sludge. Aerosol Air Qual. Res..

[35] Brugnone F., De Rosa E., Perbellini L., Bartolucci G.B. (1986). Toluene concentrations in the blood and alveolar air of workers during the workshift and the morning after. Brit. J. Ind. Med..

[36] Wishart D.S., Tzur D., Knox C., Eisner R., Guo A.C., Young N., Cheng D., Jewell K., Arndt D., Sawhney S. (2007). HMDB: The human metabolome database. Nucl. Acid. Res..

[37] Brugnone F., Perbellini L., Wang G.Z., Maranelli G., Raineri E., De Rosa E., Saletti C., Soave C., Romeo L. (1993). Blood styrene concentrations in a “normal” population and in exposed workers 16 hours after the end of the workshift. Int. Arch. Occup. Environ. Health.

[38] Knudsen J.T., Eriksson R., Gershenzon J., Ståhl B. (2006). Diversity and distribution of floral scent. Bot. Rev..

[39] Chitarra G.S., Abee T., Rombouts F.M., Dijksterhuis J. (2005). 1-Octen-3-ol inhibits conidia germination of Penicillium paneum despite of mild effects on membrane permeability, respiration, intracellular pH, and changes the protein composition. FEMS Microbiol. Ecol..

[40] McGinty D., Scognamiglio J., Letizia C.S., Api A.M. (2010). Fragrance material review on 2-ethyl-1-hexanol. Food Chem. Toxicol..

[41] Jackson J.A., Riordan H.D., Neathery S., Riordan N.H. (1997). Urinary pyrrole in health and disease. J. Orthomol. Med..

[42] Schmutzhard J., Rieder J., Deibl M., Schwentner I.M., Schmid S., Lirk P., Abraham I., Gunkel A.R. (2008). Pilot study: Volatile organic compounds as a diagnostic marker for head and neck tumors. Head Neck.

[43] Li N., Deng C., Yin X., Yao N., Shen X., Zhang X. (2005). Gas chromatography-mass spectrometric analysis of hexanal and heptanal in human blood by headspace single-drop microextraction with droplet derivatization. Anal. Biochem..

[44] Poli D., Carbognani P., Corradi M., Goldoni M., Acampa O., Balbi B., Bianchi L., Rusca M., Mutti A. (2005). Exhaled volatile organic compounds in patients with non-small cell lung cancer: Cross sectional and nested short-term follow-up study. Respir. Res..

[45] Brunner C., Szymczak W., Höllriegl V., Mörtl S., Oelmez H., Bergner A., Huber R.M., Hoeschen C., Oeh U. (2010). Discrimination of cancerous and non-cancerous cell lines by headspace-analysis with PTR-MS. Anal. Bioanal. Chem..

